# The RESearch PARamedic Experience (RESPARE) study: a qualitative study exploring the experiences of research paramedics working in the United Kingdom

**DOI:** 10.29045/14784726.2023.3.7.4.14

**Published:** 2023-03-01

**Authors:** Graham McClelland, Matt Limmer, Karl Charlton

**Affiliations:** North East Ambulance Service NHS Foundation Trust; Newcastle University ORCID iD: https://orcid.org/0000-0002-4502-5821; North East Ambulance Service NHS Foundation Trust ORCID iD: https://orcid.org/0000-0002-7873-3111; North East Ambulance Service NHS Foundation Trust ORCID iD: https://orcid.org/0000-0002-9601-1083

**Keywords:** paramedic, qualitative, research

## Abstract

**Background:**

The research paramedic position is a relatively niche role undertaken by a small number of paramedics who support, deliver and promote research. Research paramedic roles provide opportunities to develop talented researchers who are recognised as vital elements of developing a research culture within ambulance services. The benefits of research-active clinicians have been recognised at a national level. The aim of this study was to explore the experience of people who work, or have worked, as research paramedics.

**Methods:**

A generic qualitative approach underpinned by phenomenological concepts was used. Volunteers were recruited via ambulance research leads and social media. Online focus groups allowed participants to discuss their roles with peers who may be geographically distant. Semi-structured interviews expanded on the focus group findings. Data were recorded, transcribed verbatim and analysed using framework analysis.

**Results:**

Eighteen paramedics (66% female, median involvement in research six (interquartile range 2–7) years) representing eight English NHS ambulance trusts participated in three focus groups and five interviews lasting around one hour, in November and December 2021.

Six key themes were identified: starting as a research paramedic; barriers and facilitators to working as a research paramedic; research careers; opportunities; the community (support and networking); and the value of a clinical identity.

**Conclusions:**

Many research paramedics had similar experiences in terms of starting their career by delivering research for large studies, then building on this experience and the networks they create to develop their own research. There are common organisational and financial barriers to working as a research paramedic. Career progression in research beyond the research paramedic role is not well defined, but often involves building links outside of the ambulance service.

## Introduction

The research paramedic is a relatively niche role undertaken by a small number of paramedics who support, deliver and promote research within their individual settings. Research is recognised as one of the four pillars of paramedic practice in the United Kingdom (clinical, management, education and research) and is included in the College of Paramedics’ (2018) career framework. However, there are fewer paramedics working in research than any of the other pillars, and the routes to becoming a research paramedic are less clearly defined (Wood, 2012).

The National Institute for Health and Care Research (NIHR) published a review in 2016 highlighting the excellent research happening in ambulance services and illustrating a range of studies supported by research paramedics (NIHR Dissemination Centre, 2016). This report described the need to develop a research culture in ambulance services which research paramedics can champion and act as focal points for. Building research capacity and developing talented researchers are recognised as vital elements of developing a research culture within ambulance services, and both are key aspects of the research paramedic role (Siriwardena et al., 2010).

The need for clinical academics in nursing, midwifery and the allied health professions (NMAHPs) is clear (AUKUH Clinical Academic Roles Development Group, 2016), and the benefits of research active clinicians are well recognised (Chief Allied Health Professions Officer’s Team, 2017). Despite this, there is a paucity of literature discussing or describing the research paramedic role (McClelland, 2013; Whitley & Wilson, 2022; Wilson et al., 2022).

Due to the lack of literature and the small number of research paramedic roles, the routes into research are less clear than those into other areas of practice, and there is little information about the research paramedic role to inform potential applicants. Research paramedic roles can be temporary, and when a project or study comes to an end, the experience and learning that has been accumulated by the research paramedic can be lost as the paramedic returns to their original role or moves into a different position.

### Aim

To explore the phenomena of the research paramedic by capturing insights from research paramedics who are working, or have worked, in research and have a lived experience of the role.

## Methods

This study is reported using the consolidated criteria for reporting qualitative research (COREQ) guidelines (Tong et al., 2007).

A pragmatic, generic qualitative paradigm (Caelli et al., 2003; Cooper & Endacott, 2007; Williams, 2012b, pp. 100–103) was used. The study was underpinned by phenomenological methods suitable for exploring the lived experience of research paramedics (Balls, 2009; Rodriguez & Smith, 2018).

### Setting and participants

Paramedics who currently work, or have worked in the last five years, as research paramedics in NHS ambulance services in England or Wales.

### Sampling and approach

Participants were purposively sampled for maximum variation, which in this case was interpreted as representation from as many different settings as possible. We reached the target population via research and development managers at each ambulance trust identified from the National Ambulance Research Steering Group (NARSG), social media and the authors’ own contacts. Potential participants were asked to contact the research team, who provided study information via email. Participants received a £20 Amazon gift voucher to compensate for their time.

### Sample size

The study aimed to conduct three focus groups, followed by up to six interviews for participants who did not attend the focus groups. The recruitment target was 12–24 participants. Data saturation was not explicitly sought, rather the sample size was a pragmatic decision based on the resources available and the small target population (Braun & Clarke, 2021).

### Data collection

Focus groups (Williams, 2012a, p. 82) were used to allow participants to discuss their roles, share and compare experiences and consider what would be useful for people to know about the research paramedic role. This method allowed research paramedics working in different geographical locations and clinical settings to discuss their roles with peers with similar experiences.

Semi-structured interviews (Williams, 2012a, p. 81) were offered to allow volunteers who were unable to attend the focus groups the opportunity to participate.

Focus groups and interviews were conducted online via Microsoft Teams or Zoom, due to the geographical distribution of participants and COVID-19 restrictions. A topic guide was used to provide structure to the focus groups and interviews, which evolved over the course of the study (see Supplementary 1). Focus groups and interviews were audio-recorded, transcribed verbatim and anonymised for analysis. Field notes were taken by the research team during focus groups and interviews.

### Consent

Participants provided written informed consent.

### Data analysis

Analysis was performed using 5-stage framework analysis (Lacey & Luff, 2007), allowing systematic data analysis with clearly defined stages.

All authors initially analysed one focus group and one interview, then compared notes and themes; this formed the basis for the coding framework that was applied to the whole dataset by one author (GM). Themes were developed from the a priori topics included in the topic guide and emergent themes from the data.

### The research team, risk of bias and reflexivity

GM is a North East Ambulance Service (NEAS) research paramedic currently doing a post-doctoral fellowship at Newcastle University focused on emergency stroke care, and has worked in research for over 10 years. ML is a research paramedic with NEAS currently working on the PARAMEDIC3 trial, is also completing an applied research collaborative (ARC) fellowship and has worked in research for five years. KC is a research paramedic with NEAS, is currently completing a PhD with Northumbria University focused on cardiac arrest management and has worked in research for seven years.

As the authors work as research paramedics, efforts were made to acknowledge bias from their perspectives as researchers working in the same role and field. This was addressed by summarising key points at the end of focus groups / interviews, sending summaries to participants for member checking, reflecting on the potential influence of personal views during data analysis and discussing the analysis with a non-paramedic researcher.

### Patient and public involvement

There was no patient or public involvement in this study.

## Results

Eighteen paramedics (66% female, median involvement in research six years (interquartile range 2–7)) who worked for, or had worked for, eight English NHS ambulance trusts participated in three focus groups (mean duration 75 minutes) and five interviews (mean duration 50 minutes) in November and December 2021. No participants dropped out or withdrew from the study. All focus groups and interviews were facilitated by GM with support from ML.

Six key themes were identified:

1. starting as a research paramedic;
2. barriers and facilitators to working as a research paramedic;
3. research careers;
4. opportunities;
5. the community (support and networking); and
6. the value of a clinical identity.

### Theme 1: starting as a research paramedic

Many participants described similar experiences of how they started as a research paramedic, often through involvement in externally funded studies such as PARAMEDIC (Perkins et al., 2015), AIRWAYS2 (Benger et al., 2018) and PASTA (Price et al., 2020). Most participants undertook the role of research paramedic with little or no previous research experience:

*For me starting the role, it was just a huge learning curve, I didn’t expect it to be easy and straightforward, but I didn’t realise actually how much was involved in becoming a research paramedic. So for me it’s been a whole new experience being a paramedic for so long and then going into research.* (Participant 11)

The initial research paramedic role focuses on research delivery rather than development or design. The hands-on, practical, problem-solving nature of this work was viewed as interesting and valuable by participants.

Many participants were involved in research in addition to working clinically prior to being a research paramedic. Some participants described self-funding qualifications and courses to prepare them for working in research. Initial research paramedic roles were often temporary and lacked job security.

Modern university-based paramedic training was viewed as advantageous in terms of exposure to research and academia, but practical ambulance experience and service were also valued:

*I find now the paramedics who’ve gone through the university route, they are more engaging with the research, they understand it a bit more, and it’s almost as though the other paramedics are a bit frightened of it, it’s a little bit alien to them.* (Participant 11)

The educational qualifications needed for a research paramedic role were discussed at length. Participants agreed a BSc (level 6) qualification was sufficient to begin a career as a research paramedic but a Master’s (level 7) was useful to progress as a clinical academic.

Participants identified a lack of information for paramedics looking to move into research. The absence of a standard research paramedic definition and the small number of people working in the role made finding out about the role challenging. Personal contacts or links with local ambulance service research departments were seen as good ways to find information for people interested in the role.

Participants discussed their motivations for getting into research; these included the opportunity to improve care for a large number of patients, interest in specific diseases/presentations and career longevity and development. Research was also viewed as a way of moving away from clinical duties for people disillusioned with frontline clinical practice:

*there was quite a growing frustration in that whenever I was looking for an answer or justification as to why we did stuff the way we did, other than we’ve always done it that way, there was none. So you scratch around and looking at papers and looking at other stuff and finding that actually, everything seems to be just based on consensus because no one actually knew the answer. So increasingly, there are two things really. One of them is I got a bit frustrated by the fact that you didn’t know if you were doing the best thing for the patient and obviously over time, you realise we are not doing the best thing for patients by doing certain treatment methodologies. Secondly, I started realising, as a paramedic on the back of a bus, generally you’re seeing one patient at a time, and that’s quite desirable. But actually, there are opportunities to have a wider positive impact for people.* (Participant 4)

### Theme 2: barriers and facilitators to working as a research paramedic

A number of barriers to moving into and sustaining a research paramedic role were identified. One of the common barriers discussed was the negative financial impact, largely due to the loss of unsocial hours payments. This financial consideration meant that an individual’s particular circumstances (age, baseline salary, dependants) may influence whether a research paramedic role was seen as attractive or even a viable option for paramedics considering it:

*The financial stuff is a bit of a barrier. But I think we just need to feel quite competent in where this particular role sits at the moment. When I took this, I had to drop the 25% unsocial, so it was a pay cut to do it, even though the bandings don’t change or anything. But the way I sort of sold it to myself was, it will progress my career, there’ll be other results of doing this, there will be better financial opportunities in the future.* (Participant 5)

The temporary nature of many research paramedic roles was seen as both a barrier and facilitator. Some participants viewed the temporary nature of roles as a good thing if people wanted to try research but did not want to commit to it in the longer term. Other participants viewed the temporary roles as a barrier for staff valuing stability and longer-term security.

Research was viewed as not being a priority and less valued than other areas of practice by ambulance trusts, which was perceived as a barrier to working as a research paramedic especially when compared to other potential career options:

*becoming a senior paramedic team leader or advanced paramedic is much more in your face when it comes to options that you have as a paramedic. And probably also going into primary care and other options like that are much more well-known than becoming a research paramedic or having a research career as a paramedic.* (Participant 1)

The nature of the day-to-day work of research was seen as a potential barrier to some paramedics, with the job viewed as desk based, involving sometimes repetitive tasks and often lacking personal contact with other staff and patients compared to clinical roles. However, the variation, opportunities and potential for long-term development afforded by working in research were seen as countering this negative perception of the role.

### Theme 3: research careers

Working as a research paramedic was compared to working in other areas of practice and the range of career trajectories open to paramedics now. Research was seen as a niche career with fewer opportunities than other areas such as critical or primary care.

The career pathway for paramedics working in research was viewed as unclear compared to other areas of practice and was regarded as still being in development, largely by the small number of paramedics already in the role who are pushing the boundaries and developing new opportunities. The recent emergence of advanced and substantive research roles was seen as a positive step for research paramedics and those considering it as a career direction:

*going back to 10 years ago, even doing pre-hospital research was really unknown. So there wasn’t those roles there. You know research didn’t get done in an ambulance trust kinda thing. Research didn’t get done in the pre-hospital world because it was deemed as too difficult. So I think the more time goes on the more we’ll get a really good track record with doing research. It might then open it up to people seeing it more of a career that’s viable.* (Participant 17)

The day-to-day work of a research paramedic was described as being focused on research delivery, involving activities such as training staff, collecting data and following up patients. Career progression was described as moving into research development and involved developing projects and designing studies:

*for most of us it started with a research paramedic post and we’ve delivered research. And for most of us, again, we’ve gone on and we’ve developed and designed our own research.* (Participant 4)

Research leadership roles, such as professorships, were discussed as a long-term career goal for people pursuing the research paramedic / clinical academic pathway.

Participants recognised that university qualifications and links become increasingly important when pursuing a career in research, and that ambulance trusts, in their present incarnation, may not be the most suitable host institutions for people pursuing this career direction. PhDs and doctorates (level 8) were seen as a goal or stepping stone on a research career. Fellowships offered by organisations like the NIHR were seen as an attractive way of completing doctoral-level training, due to the time and resources available in these schemes and the difficulty of completing doctoral studies without substantial support.

### Theme 4: opportunities

The variety of opportunities that working in research provides was a clear theme. Opportunities to develop new skills, collaborate with people from within and outside the ambulance service and influence care in a different way and at a different scale were all discussed.

Participants believed working with people outside of the ambulance service was necessary to many research projects, and that this provided career opportunities that may not have been available before working in research:

*I suppose it depends on how, I suppose how much you wanted to take those opportunities that came along. For me, starting out, doing XXXXXX, I never thought I would then go and you know, move away and work elsewhere and work at a clinical trials unit the way I did. But I was just very lucky that those opportunities came along, so I suppose that’s part of it*. (Participant 17)

The increasing need for lecturers in universities was an opportunity that was discussed, with the research paramedic role perceived as a good platform to teach students about research and improve on some of the negative experiences of research teaching that participants had experienced.

Working in research provides opportunities that paramedics working in clinical roles would find more difficult to access, such as attending training courses and conferences. These were viewed as part of the research paramedic role, rather than activities that would otherwise be undertaken in your own time, above and beyond a full-time clinical job:

*Making the most of opportunities to kind of I suppose develop even in ways that you wouldn’t necessarily expect. So I’m just thinking about, I learned about how to do infographics for XXXXXX to try make information more interesting than sending an email just with words. I suppose thinking outside the box, not necessarily going with the obvious*. (Participant 14)

Taking advantage of the opportunities provided by working in research is not always straightforward. Participants talked about the need for a trust to be research active to provide opportunities and the value of supportive colleagues who could cover your work while you pursued other opportunities.

### Theme 5: the community (support and networking)

Research departments in ambulance services tend to be small, with only a few people working in the team. Working in research can involve working alone and remotely, which could potentially feel isolating. This could be exacerbated by recent remote working practices as a result of the COVID-19 pandemic. Working as part of a small, tight-knit team was seen as different from working on the road and invoked a different sense of community.

Participants described how finding and working with other research paramedics, both within and across organisations, gave a sense of belonging to a community, which reduced feelings of isolation. Developing these internal and external networks was seen as important to working as a research paramedic:

*networking with other research paramedics, for me that was really useful on the XXXXXX trial because it was my first research paramedic post so I was a bit uncertain about how to run the study from a day-to-day basis and our regular meetings were really helpful.* (Participant 4)

Ambulance trust research departments were identified as key contacts; the College of Paramedics was highlighted as a source of information, and a small number of centres recognised as leading paramedic and pre-hospital research were also named as key players and sources of information. The small number of leading centres was identified as a potential barrier if you were unable to link in with these groups, but also as a facilitator if you did have links. Research conferences and multi-centre studies were seen as methods of developing links and networking.

Interacting with external networks was seen as an important aspect of being a research paramedic, and participants described the value in building links with people and organisations. External networking was also seen as a way to promote research in ambulance services and with paramedics:

*I’d be attending various meetings, particularly external meetings and as I sort of go back, oh you’re a research nurse, no I’m a research paramedic. So actually, for want of a better phrase, I was, not a novelty, but actually I stood out from the crowd. So it was helpful in a kind of, hopefully acquiring or gaining or developing a positive reputation and being able to develop the whole early days of networking and boundary spanning as they call it. And I kind of know people in all sorts of different areas of clinical practice. But also, I don’t know if you’ve had this, but I would be able to attend, I wouldn’t say any meeting, but I would go to cardiovascular specialist meetings or the neuro specialist meetings. So from that perspective, when you’re then the only one you do, your head is above the parapet, just by turning up and particularly turning up in a green uniform always goes down well. I shouldn’t say that, should I, but it makes you memorable. *(Participant 16)

### Theme 6: the value of a clinical identity

A clear theme that came out of the study was how research paramedics valued their clinical identity and the ‘paramedic’ part of being a research paramedic. All participants started as paramedics before they moved into research roles and saw this as a core part of their identity; it was often working as a paramedic that generated ideas or insights that were then pursued while working in research.

Maintaining their practice as a paramedic was highly valued by participants, who described how it opened doors for them and made delivering research and communicating with other ambulance staff about research easier, and how their clinical insight was useful in translating ideas originating outside of the ambulance service into workable studies. Credibility was a term that was frequently used:

*I think that dual role really helps to integrate research into clinical practice . . . So I think it gives research a clinical presence and I also think credibility as a research paramedic to those that you are trying to recruit into studies . . . I’m not ready to step away from clinical practice, I very much enjoy it . . . I really like the balance of both*. (Participant 7)

Balancing clinical and research duties was a difficulty for many participants. Operational demands were described as one of the challenges to working in research, especially in recent years when ambulance trusts have been under sustained pressure. Being a research paramedic came with a perceived loss of clinical skills, which concerned participants. This was countered by recognition that new skills were being developed. While research was seen to complement clinical practice, progressing as both a researcher and clinician was perceived as very challenging. Participants believed that pursuing an academic pathway often resulted in a loss of clinical skills and credibility:

*I think it’s really easy to become deskilled quite quickly, so I think keeping your own practice up to date can alleviate some of those fears in terms of thinking what happens if I gotta go back on the road next week kind of thing*. (Participant 17)

## Discussion

This study describes themes collected from interviews and focus groups with research paramedics from across England. Six key themes are presented, encompassing topics including starting as a research paramedic, what the role entails, developing a career as a research paramedic and how participants view their dual role and identity. The themes reported here are similar to those described by allied health clinician researchers from Australia (Brandenburg & Ward, 2022). Some of the themes identified in this study, such as the need for job security and the benefits of networking, are similar to those reported in studies of NMAHP clinical academics (Roddam et al., 2019).

There has been little published about research paramedics (McClelland, 2013; Whitley & Wilson, 2022; Wilson et al., 2022), so this study adds to the body of evidence and hopefully makes the role more accessible to paramedics considering research as a career. A lack of information about research opportunities and clinical academic careers has been identified as a barrier to developing NMAHP clinical research (Jones & Keenan, 2021).

Participants were largely positive about the research paramedic role and expressed that it was worthwhile, gave them opportunities to develop professionally and allowed them to influence patient care. The desire to improve patient care was a key motivating factor for many participants, which is similar to the motivation reported in other clinical academic researchers (Trusson et al., 2019).

While routes into the research paramedic role were reasonably consistent within the study (part time, temporary, externally funded) and examples were given of more senior roles emerging in recent years, the longer-term career pathway for paramedics wanting to pursue research remains unclear in ambulance services. There are models of clinical academic career pathways such as the integrated clinical and practitioner academic programme promoted by Health Education England (HEE) and the NIHR (HEE-NIHR, 2022), but examples of paramedics reaching the latter stages of these programmes, and senior academic/research roles, are rare. Difficulties moving into senior clinical academic roles have been reported in other studies of NMAHP clinical academics (Jones & Keenan, 2021; Trusson et al., 2019).

Participants expressed how networking within ambulance services and outside was a key part of the role and how they acted as ambassadors for their services and the profession. This was viewed as a positive part of the role by most participants, who enjoyed the chance to raise the profile of the profession and ‘*to cheerlead the paramedic profession and the ambulance profession in those different spheres of healthcare*’ (Participant 18).

While participants acknowledged that research involved different skills and ways of working which could lead to feelings of isolation, there was a strong feeling of membership of a research community, almost a tribe within the wider paramedic tribe. Support from the wider research community was valued and contrasted with how participants perceived research as being valued by ambulance trusts. Jones and Keenan (2021, e196) stated that ‘perceptions of the value of academic NMAHPs in clinical and academic organisations have not developed as fast as the professions themselves’, illustrating that this perceived lack of value is not unique to the ambulance service.

### Strengths and limitations

This study included participants from most UK ambulance trusts and while there are no robust figures on the number of research paramedics employed during the study timeframe, the authors suggest that this sample is reasonably representative of research paramedics due to the small number of staff involved in ambulance service research.

No participants from Northern Ireland, Scotland or Wales were included in the study, which may limit the transferability of any findings to these countries, however the experience of working as a research paramedic should not be dramatically different across the United Kingdom, due to the similar ambulance services and research structures. The findings from this study reflect the current UK model, which is paramedic-led care underpinned by level-6 (BSc degree) study as a baseline, and with a growing research culture. The findings may not be applicable in different settings with different educational requirements.

Other limitations include the potential for bias to be introduced by the authors, all of whom work as research paramedics, and the pre-existing relationships between authors and participants. Members of the research team have published and presented on the research paramedic role, so participants may have had foreknowledge of the aims and intentions of the study in addition to the information in the participant information sheet.

### Recommendations for professional practice and future research

Many participants began their research careers working on multi-centre, externally funded trials, but these come along infrequently and often with a limited number of roles. Consequently, these are an unreliable route for paramedics wanting to explore research, so other more consistent routes into research need to be developed and promoted.

The financial barriers to working in research identified in this study have been recognised elsewhere (Brandenburg & Ward, 2022; Trusson et al., 2019). Many NMAHP clinical academics have years of clinical experience before moving into research (Jones & Keenan, 2021), resulting in higher salaries or positions at the top of their banding. If the paramedic profession wants to make research a viable career option and attract and retain individuals who will go on to lead research and improve care, then these financial barriers need to be addressed.

While this study focused on research paramedics, future research should incorporate the views of research managers, directors and other key roles working in ambulance services. Research managers have been recognised as being key to providing a supportive environment for clinical academics to work in (Roddam et al., 2019). Senior support, understanding of the research paramedic role and recognition of the benefits it brings are vital if paramedic research is to continue to develop.

Hybrid roles, where paramedics can maintain clinical practice and links and also develop as researchers and academics, are necessary and are the well-established model for physicians. These roles require the ambulance service to recognise the value of the clinical academic and may require partnerships with universities. The establishment of senior clinical academic paramedics, and more research paramedics becoming professors, will establish not only a recognisable career pathway but also provide mentorship and guidance for novice research paramedics.

## Conclusions

The research paramedic role appears to be valued by those who have undertaken it, and offers opportunities to develop and a different way to influence patient care. Many research paramedics had similar experiences in terms of starting their career by delivering research for large studies, then building on this experience and the networks they create to develop their own research. There is a community of paramedics involved in research, which was seen as supportive and beneficial. There are common organisational and financial barriers to working as a research paramedic. Career progression in research beyond the research paramedic role is not well defined but often involves building links outside of the ambulance service.

## Acknowledgements

The authors would like to thank Emma Burrow from NEAS for her support with this project.

## Author contributions

All authors were involved in the development of this study. KC led the funding bid. GM and ML facilitated the focus groups and interviews. All authors were involved in the analysis of the data. GM drafted the article, which was reviewed and approved by all authors. GM acts as the guarantor for this article.

## Conflict of interest

GM is the editor-in-chief of the *BPJ*.

## Ethics

This proposal was developed with input from the North East and North Cumbria Research Design Service. Health Research Authority (HRA) approval was secured (IRAS ID 298311). Research Ethics Committee (REC) approval was not necessary, as this was a staff-focused study. The study was adopted onto the NHS CRN portfolio.

## Funding

This study was funded by a College of Paramedics small research grant.
